# The Disestablishment of the Poor House

**Published:** 1891-06-13

**Authors:** 


### The Hospital

A Weekly Journal of

Science, Medicine, Nursing, and Philanthropy.

*For Hospitals, Asylums, Infirmaries, Dispensaries, Medical Practitioners, Students, Nurses, and the Charitable Public.*

Vol. X.—No. 246.] [June 13, 1891.

### The Disestablishment of the Poor House.

After ten years of "crying in the wilderness," Canon Blackley is having his reward in the attention which public men of weight and influence are giving to his proposals for national pension assurance. The poor of this country are a standing reproach and grief to many of their more prosperous neighbours. Whatever be the causes that have gradually produced the present state of things, there is no manner of doubt that the average man who occupies the position of a day labourer leads a life of exhausting and hopeless toil, and generally spends his old age as a pauper, dies a pauper's death, and is buried in a pauper's grave.

Clergymen and doctors are almost the only persons of education who see much of the poor, especially in country places. The landed proprietor seldom knows the cottagers on his estate even by name; and the farmer, as a rule, has no pretence whatever to be called an educated man. The clergyman and the doctor agree in thinking the poverty of the poor an unmixed evil. The farmer does not think so: he prefers that his labourers shall be poor. He knows that so long as they are dependent upon him for food from week to week, he can rule them more or less successfully. But he knows, also, that if they had a little money in hand, or a piece of garden ground or grass land by means of which they could manage to live for a few weeks or months occasionally without other employment, they would be much less yielding than they now are to his petty tyrannies and vacillating rule. The average farmer is fully conscious of his own want of ruling capacity; and he therefore desires that those whom he is called upon to govern shall be endowed with as little resisting power as may be compatible with life and the strength to work. Other employers of labour share to some extent the farmer's ignorance, prejudice, and incapacity for authority. We have chosen the farmer, however, as our typical employer of labour, because he deals with the lowest and worst-paid class of labourers; and therefore any scheme of national insurance which is such as can be applied to farm labourers may almost as a matter of course be applied to any other class.

The point of view here taken is that of the educated clergyman and doctor in country places. The clergyman is impressed with the dense and stolid ignorance of the labourer; with the dullness of his life and the coarseness of his pleasures; with his insensibility to religion and morals; and with his total incapacity to take any share in the support of religious institutions or of educational and philanthropic movements. The country labourer, in fact, from the clergyman's point of view, is not a man but a child; not a helper but a person needing to be helped; not a source of strength but a burden to be carried; a dreary, dull, and hopeless kind of social institution which must be borne with as best it may, and carried through life with as little trouble and friction to other people as circumstances will permit.

The doctor sees the labourer from another educated standpoint: he sees him associated with a half-fed wife, and many hungry and ill-clothed children; in a poor and insanitary cottage; much in debt to the grocer and the shoemaker; and quite unable to pay the smallest conceivable doctor's bill. But he sees the whole race of labourers in the few individuals he has personal dealings with, and he thinks of the mass becoming poorer in physique and duller in intelligence from generation to generation. The doctor probably resents this state of things much more than the parson. His physiological instincts are wounded by it, and his pocket is grievously injured. If he be a patriot, as most doctors are, he is filled with indignation at the physical and mental degeneration of his fellow-countrymen. If he be a historical student, he thinks with dismay of the possibility that another Crecy, or an Agincourt, or a battle of Hastings might some day be the arbiter of England's destiny. The "brave yeomen" of early English story are now replaced by a set of stupid and incapable tenant farmers; and the stubborn and unconquerable peasantry are reduced to such a hopeless level that they have neither courage nor physical energy even for a battle in defence of their own homes. The labourer has an infinity of ills to endure throughout his life; but perhaps the most heart-breaking of all the misfortunes which are his by sad inheritance is the inevitable certainty that his life of ceaseless toil will end in the dreary prison of the workhouse. We cannot be too thankful that he is mostly ill-educated and stupid; if he were otherwise, suicide in ten thousand distressing forms would be as common in our towns and villages as the changes of the weather.

Canon Blackley, Mr. Joseph Chamberlain, and others are elaborating a scheme of national pensions for labourers. It is a great undertaking as well as a new departure, and we must not expect very rapid progress to be made. But we may be permitted, notwithstanding, to hope for reasonably early results; not only because Canon Blackley's scheme has been before the world for some years, but also because the Royal National Pension Fund for Nurses furnishes, on a small scale, an example of what may be done by workers in other fields. It is thought best that national insurance shall be tried first on a voluntary basis. By way of experiment, this is no doubt desirable. The success of the Royal National Pension Fund encourages the hope that a properly-constructed scheme would meet with similar success among other workers. But it must be remembered that with all the inducements of liberal bonuses and small working expenses held out to nurses, and all the energy and experienced capacity which have been at the disposal of the National Pension Fund from its foundation until now, not more than one in every fourteen or fifteen trained nurses has been induced to provide for old age by means of its generous help. Whilst, therefore, we would rather see a scheme of national insurance tried voluntarily than not tried at all, we cannot but hope that even the workmen themselves will speedily be educated to the incalculably greater advantages of compulsion.

The pressing practical question with many is this: Can young workmen afford to lay by in youth a sum which, when placed out at interest, will furnish them with a small competence in old age? The young farm labourer certainly can; and he is the poorest of all our toilers for weekly wages. We speak from an extensive knowledge of facts. There is no doubt whatever that the average farm labourer from the age of seventeen to the age of twenty-five could lay by at the least three pounds a-year if he were compelled to do so. Of course, marriage would be prohibited before the age of twenty-five. There is no reason why, if the labourer were to set apart three pounds a year, his employer should not be made to increase the sum by one-half, that is to raise it to four pounds ten. For whatever the employer paid in this way would result in a diminution of poor rates, which would compensate him ten-fold in later life. With accumulated interest, this would give a capital sum of nearly forty pounds for each farm labourer of twenty-five years of age. Starting with an initial forty pounds, the State would be a very sorry financier if it could not elaborate a modest pension of six shillings a-week for every man who lived to reach sixty. The certainty of such a pension at sixty would be like pouring fresh life blood into the arteries of a soldier dying of his wounds. The whole labouring population would be quickened and vitalised with a new hope. No one who has not personal knowledge of the poor can have any understanding of the terrible nightmare in the shape of poverty and the looming workhouse, that crushes out all hope from their daily lives. The ugly but much less terrible nightmare of a despairing population is now beginning to be felt by men in authority. The evil to be dealt with is gigantic in its proportions, and hoary with unvenerable age. If ever it is justifiable to urge haste upon statesmen, it is justifiable in such a case as this. England is ashamed of the poverty and slavery of her millions of toiling children; and the lives of those toiling millions themselves are embittered by the sight of a social happiness in which they have no part, and of a national prosperity in which they do not share. Our one prayer for our rulers should be that their consciences may be made more tender, and that their governing capacity may be speedily exalted and increased. We could almost wish that those in authority might never know another happy hour until they have taken to heart the question of the labourer's wrongs, and resolved upon the disestablishment of the poor-house.

